# Optimizing event-based neural networks on digital neuromorphic architecture: a comprehensive design space exploration

**DOI:** 10.3389/fnins.2024.1335422

**Published:** 2024-03-28

**Authors:** Yingfu Xu, Kevin Shidqi, Gert-Jan van Schaik, Refik Bilgic, Alexandra Dobrita, Shenqi Wang, Roy Meijer, Prithvish Nembhani, Cina Arjmand, Pietro Martinello, Anteneh Gebregiorgis, Said Hamdioui, Paul Detterer, Stefano Traferro, Mario Konijnenburg, Kanishkan Vadivel, Manolis Sifalakis, Guangzhi Tang, Amirreza Yousefzadeh

**Affiliations:** ^1^IMEC, Eindhoven, Netherlands; ^2^IMEC, Leuven, Belgium; ^3^Department of Dependable and Emerging Computer Technologies, Delft University of Technology, Delft, Netherlands; ^4^Department of Computer Architecture and Embedded Systems, University of Twente, Enschede, Netherlands

**Keywords:** event-driven, neuromorphic, depth-first, spike-grouping, sensor fusion

## Abstract

Neuromorphic processors promise low-latency and energy-efficient processing by adopting novel brain-inspired design methodologies. Yet, current neuromorphic solutions still struggle to rival conventional deep learning accelerators' performance and area efficiency in practical applications. Event-driven data-flow processing and near/in-memory computing are the two dominant design trends of neuromorphic processors. However, there remain challenges in reducing the overhead of event-driven processing and increasing the mapping efficiency of near/in-memory computing, which directly impacts the performance and area efficiency. In this work, we discuss these challenges and present our exploration of optimizing event-based neural network inference on SENECA, a scalable and flexible neuromorphic architecture. To address the overhead of event-driven processing, we perform comprehensive design space exploration and propose spike-grouping to reduce the total energy and latency. Furthermore, we introduce the event-driven depth-first convolution to increase area efficiency and latency in convolutional neural networks (CNNs) on the neuromorphic processor. We benchmarked our optimized solution on keyword spotting, sensor fusion, digit recognition and high resolution object detection tasks. Compared with other state-of-the-art large-scale neuromorphic processors, our proposed optimizations result in a 6× to 300× improvement in energy efficiency, a 3× to 15× improvement in latency, and a 3× to 100× improvement in area efficiency. Our optimizations for event-based neural networks can be potentially generalized to a wide range of event-based neuromorphic processors.

## 1 Introduction

Neuromorphic processing is an emerging field of computer architecture that draws inspiration from biological brains. It offers the potential for natural signal processing with low latency and reduced power consumption. The current mainstream neuromorphic processor architectures employ bio-inspired and non-conventional techniques such as asynchronous (Lines et al., 2018), analog (Rubino et al., 2023), in/near-memory (de los Ríos et al., 2023), data flow (Moreira et al., 2020), and event-driven (Yang et al., 2021) computing to achieve these goals. However, despite their innovative approaches, the existing neuromorphic processors are still unable to rival the area efficiency and performance of conventional deep-learning accelerators in practical market-dominated applications (Christensen et al., 2022). Neuromorphic solutions suffer from large memory overheads for event-based neural network processing. This overhead results from the fragmented memory architecture used for near-memory processing and the need to store the neuron states. For example, deploying a neural network with approximately 126k parameters (which is equivalent to 1 megabyte of storage when using 8-bit precision) on a cutting-edge neuromorphic chip will require around 200 megabytes of memory (Ceolini et al., 2020). This is 100 times more memory usage than a traditional deep-learning accelerator assuming the worst-case scenario of 100% overhead memory usage for other variables. As a result, this solution is quite expensive and requires a large silicon area. Moreover, event-driven processing in the neuromorphic chips results in a significant overhead to process a single event, which reduces power efficiency, especially for applications without excessively high activation sparsity. Consequently, Achieving competitive performance and area efficiency in neuromorphic systems remains challenging.

Event-driven data-flow computation, inspired by the sparse spiking activities in cortical networks (Wolfe et al., 2010), is the primary computing paradigm in the majority of neuromorphic processors (Nilsson et al., 2023). It takes advantage of the sparsity in neural activation (spikes) to skip redundant operations. Additionally, the data-flow processing allows the data to move directly to the location where it will be consumed instead of an intermediate shared memory, reducing the data-movement cost (Carkci, 2014). However, fine-grained event-driven processing per single spike can introduce significant overheads if the activity of the network is not sufficiently sparse, reducing the efficiency of neural network computation. Recent neuromorphic solutions attempt to reduce the overhead ratio by increasing the information encoded in the events utilizing sparse temporal encoding (Kheradpisheh and Masquelier, 2020; Guo et al., 2021) or graded spikes (Moreira et al., 2020; Orchard et al., 2021; Yan et al., 2021). Nevertheless, the overheads, partially introduced by encoding/decoding individual events, memory accesses, address calculations, and computing paradigm alterations (Hugues and Petiton, 2010), have yet to be explored and studied in existing neuromorphic systems. Therefore, there is a need for comprehensive design space exploration in optimizing event-based neural network computation on neuromorphic processors.

Near- or in-memory processing serves as another dominant design principle of neuromorphic processors to enable low-latency and energy-efficient computations. Near-memory processing involves storing data near the processor to minimize the cost of data movement, which is mostly practiced by using smaller and distributed blocks of memory near the processing elements of the chip (Akopyan et al., 2015; Lines et al., 2018; Frenkel et al., 2020; Stuijt et al., 2021). However, using a fragmented set of memories significantly reduces mapping efficiency (Jain et al., 2022). To maximize the advantage of near-memory processing in event-driven data-flow computation, most neuromorphic solutions map the complete neural network with network parameters (weight, bias, etc.) and all neural states on a multi-core system [also known as spatial mapping (Xue et al., 2023)]. This demands large neural state memory and a flexible parameter/state memory ratio. The constraints imposed by the fragmented set of memories make it difficult for the mapping algorithm to efficiently use the limited (and expensive) on-chip memory, thus reducing the area efficiency of the platform. To resolve this, recent neuromorphic architectures use unified memory space within their neuro-synaptic cores to leverage flexible mappings (Moreira et al., 2020; Orchard et al., 2021; Yan et al., 2021), resulting in less fragmented memories. Additionally, the use of lower precision data types for weights and neuron states can further reduce memory usage. Yet, for the widely used convolutional neural networks (CNNs), the neural state memory cost increases quadratically with the spatial resolution of the input tensor, resulting in low area efficiency for event-driven convolutions.

This work presents our exploration on optimizing event-based neural network processing for a neuromorphic architecture. We seamlessly integrate optimization concepts of data reuse (Sze et al., 2017) and process scheduling (Waeijen et al., 2021; Mei et al., 2023) borrowed from deep-learning accelerator architectures into the brain-inspired neuromorphic design principle. Thereby, we propose the spike-grouping method to process spikes in batches, which helped reduce the total energy consumption and latency of event-based processing. Additionally, we present the event-driven depth-first convolution, which significantly lowers the total memory requirements and the processing latency of CNN inference on a neuromorphic processor with event-driven data-flow computation. To perform a comprehensive exploration, we benefit from the flexibility of the SENECA neuromorphic processor (Tang et al., 2023b) to conduct a series of targeted experiments. These experiments provided valuable insights into various optimization techniques and leveraged hardware-algorithm co-optimizations. We then benchmarked and quantified the effects of our optimizations in the event-driven neural network processing pipeline of SENECA against other state-of-the-art neuromorphic and conventional accelerator solutions (Esser et al., 2015; Lines et al., 2018; Blouw et al., 2019; Yan et al., 2021); using accuracy, energy, latency, and area efficiency as metrics. The proposed optimizations result in a 6 to 300× improvement in energy, a 3 to 15× improvement in latency, and a 3–100× improvement in area efficiency. These results provide new insights into optimizing event-driven computation and pave the way for the evolution of event-based neuromorphic processing.

In the rest of this paper, we first give an overview of the SENECA architecture. Then, we provide a detailed explanation of our proposed optimizations for event-based neural network processing. Following this, we present the results of our experiments in three sections. The first result section focuses on the insights developed from the hardware-algorithm co-optimization exploration. The second result section reports the results with the *gesture recognition* (Ceolini et al., 2020) and *MNIST handwritten digit recognition* (Deng, 2012) tasks when comparing our event-driven depth-first convolution approach with the current state-of-the-art. Finally, the Prophesee 1M Pixel automotive detection dataset (Gen4) (Perot et al., 2020) was used to conduct an in-depth benchmarking analysis for object detection. In the end, we conclude this work with discussions of the current limitations, future works, and impact.

## 2 Methods

### 2.1 SENECA event-driven neuromorphic architecture

**Figure 1:**
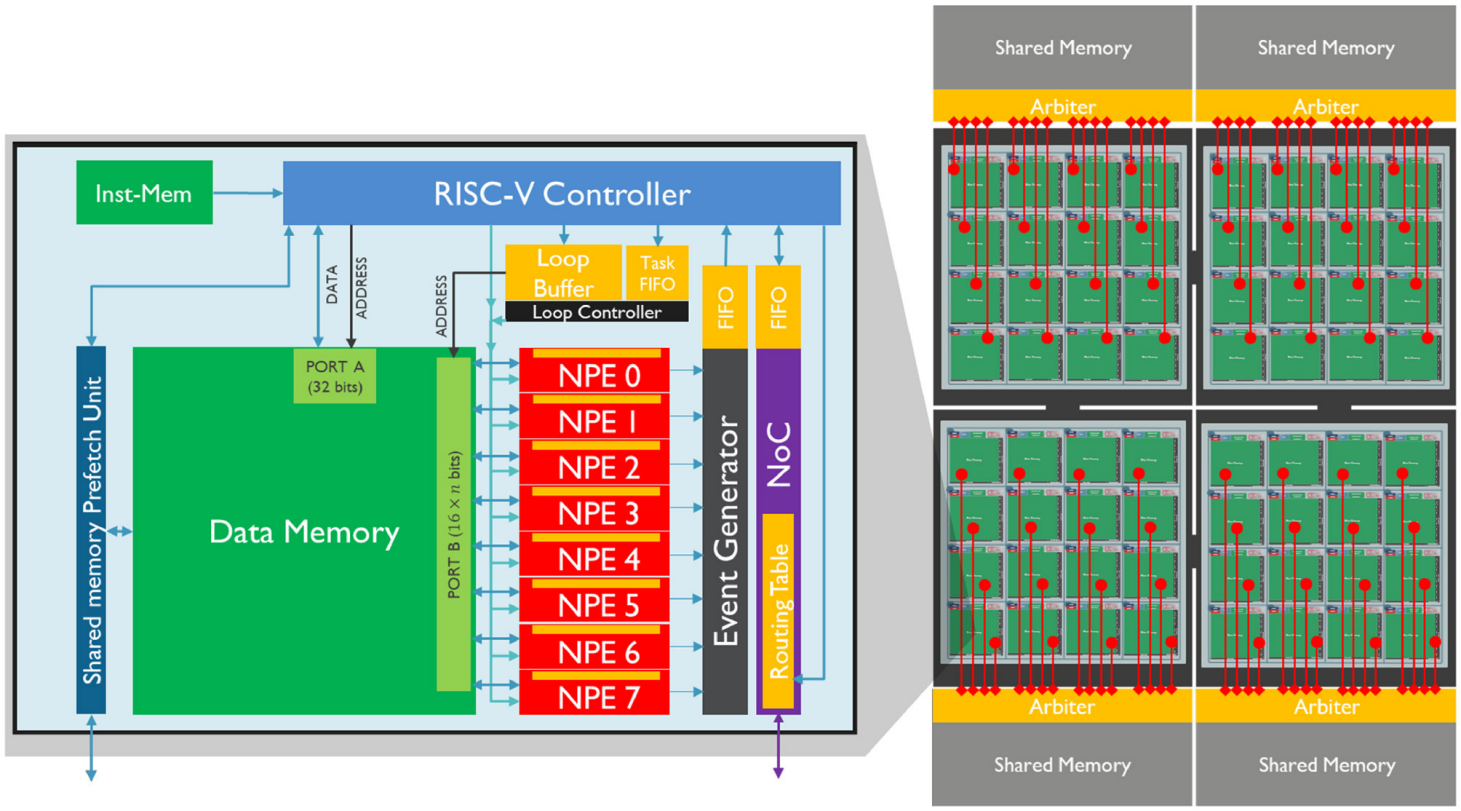
**Left**: A core of SENECA and its internal pipeline. It contains a double controlling system (RISC-V and Loop Buffer), 8 Neuron Processing Elements (NPEs), an Event Generator, a Network on Chip (NoC), and a Share-Memory Prefetch Unit (to access the shared memory). The orange blocks are the register-based memories, and the green blocks are the SRAM memories. **Right**: Four interconnected clusters, each containing 16 SENECA cores (connected through the NoC) and one shared memory (MRAM or HBM).

SENECA is a programmable digital neuromorphic processor that is capable of performing a wide range of tasks. The processor is designed with a scalable number of cores, as depicted in Figure 1, with each core consisting of a data memory, a flexible controller (RISC-V), a dedicated controller (loop controller), an event capture unit, a number of neuron processing elements (NPEs) that operate in a vector-like fashion, and a programmable Network on Chip (NoC) which facilitate the event communication among the cores.

Although the figure shows only eight NPEs in a core, the processor allows for the configuration of the number of NPEs, with up to 128 NPEs per core. The NPEs are hardware functional units that are time-multiplexed to perform neuron activity computations, providing a balance between parallelism and configurability.

Each NPE is connected to a high-bandwidth SRAM data memory (16 bits for each NPE) and has a register file (RF) with 64 16-bit words that can be used for computation. This improves energy efficiency as the access energy cost is smaller than the SRAM memory. When in computation mode, all NPEs work in lock-step mode, executing the same instruction at any given cycle similar to a single instruction, multiple data (SIMD) operation.

As a neuromorphic platform, SENECA generates spike outputs through the NPEs when they meet certain conditions according to the workload. These spikes are then processed by the event capture unit, which converts the input spike vector into address event representation (AER) format (Yousefzadeh et al., 2017). The event capture unit sends an interrupt to the RISC-V controller for further processing whenever a new spike is generated. The spikes that are generated can either be consumed in the same core or transmitted to another core through the NoC. The NoC delivers the event to the destination core based on the content of its routing table, which can change dynamically by RISC-V.

The RISC-V controller decides which operations should be executed on the NPEs depending on the workload scheduling. The loop controller coordinates the time-multiplexing of NPEs and the address generation for data memory access. It dispatches microcodes to the NPEs, enabling the processing of events. Each microcode is invoked to handle a specific type of event, such as neuron updates, threshold evaluations, or data conversions. For a more in-depth review of the SENECA architecture, please refer to Yousefzadeh et al. (2022), Tang et al. (2023a,b).

### 2.2 Event-driven neural processing on SENECA

In order to optimize the processing of sparse data flows between layers of neurons, SENECA executes event-driven neural processing. There are different types of events, where each type triggers a specific set of computations, such as binary spikes produced by spiking neurons, non-zero activations generated by the ReLU activation function, and inter-core synchronization signals representing the end of the time step or data frame. In general, event-driven processing for neural networks includes three phases:

- **Event reception**: Unpack the event and prepare for neural processing based on the information carried by the event and the recipient neurons.
- **Neural processing**: Execute neurosynaptic computations and update neural states.
- **Event transmission**: Pack the generated spikes in one event packet and multi-cast it to the destination core(s).

**Figure 2:**
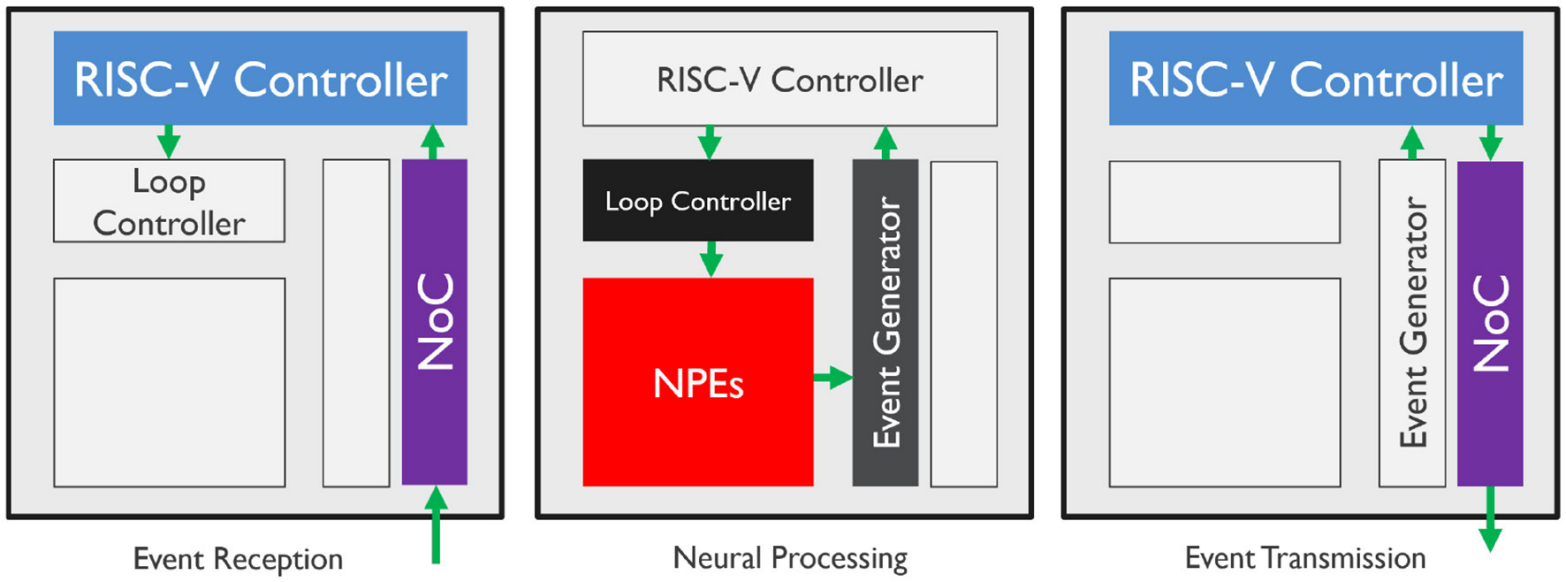
Hardware processing flow of event-driven paradigm components. The colored blocks demonstrate the active elements of the SENECA core. The components can execute asynchronously and in parallel.

Figure 2 illustrates the data flow and the hardware components involved for each phase of the event-driven processing pipeline. During neural network computation, an event received from the NoC wakes up the RISC-V controller in a SENECA core and triggers the event-reception phase. According to the decoded event, the event-receiving function determines the type of neural processing required and defines a set of executable tasks (which are represented by micro-code to be executed at the NPEs). The loop controller receives the tasks and controls the time-multiplexed neural processing steps in the NPEs. The loop controller operates asynchronously with the event-receiving functions, allowing for accelerated and parallelized event processing. If a task execution involves event generation from neural states, the event generator collects non-zero outputs from NPEs and packs them as AER events. These events then wake up the RISC-V controller and trigger the event-transmission phase, which encodes and packages the AER events as compressed network event packets. Finally, the network event packet is sent to the destination cores through the Network on Chip (NoC) for further processing. Neural processing through the loop controller can work in parallel with the event reception/transmission processes since the loop controller can orchestrate the neural processing independently from the RISC-V controller.

For inference of the neural networks that are trained with time-step (synchronous), we need another type of network event packets to synchronize cores and signal advancement in time-step. For example, during the inference of an event-based fully connected (FC) neural network layer, two types of events are involved: non-zero neuron activation events and a synchronization event at the end of each time step. The synchronization event marks that all required input information for the current time step has been received.

- When an **activation event** is received, an *event-integration* task is executed. The *event-integration* task multiplies the activation value with the weight vector and integrates the results to all neural states.
- When a synchronization event is received, an *event-generation* task is executed. The *event-generation* task applies the activation function (e.g., ReLU) to neural states and possibly generates non-zero activation events.

We can map multiple FC layers of a neural network to a single SENECA core and thus apply the same event-driven processing for each layer. When having multiple layers mapped on a core, **event-transmission** from one layer directly passes events to the **event-reception** of the succeeding layer on the same core without involving the NoC.

It is worth noting that this paper exclusively utilizes spikes with graded values. These spikes are produced by applying the ReLU activation function to the neurons at the end of each time step. Previous research (Tang et al., 2023b) has demonstrated that integrating graded spikes results in minimal energy overhead while achieving high accuracy in our tasks. The use of spikes with graded values is gaining acceptance in digital neuromorphic processors, where each spike is encoded into AER format.

### 2.3 Event-driven depth-first convolution

**Figure 3:**
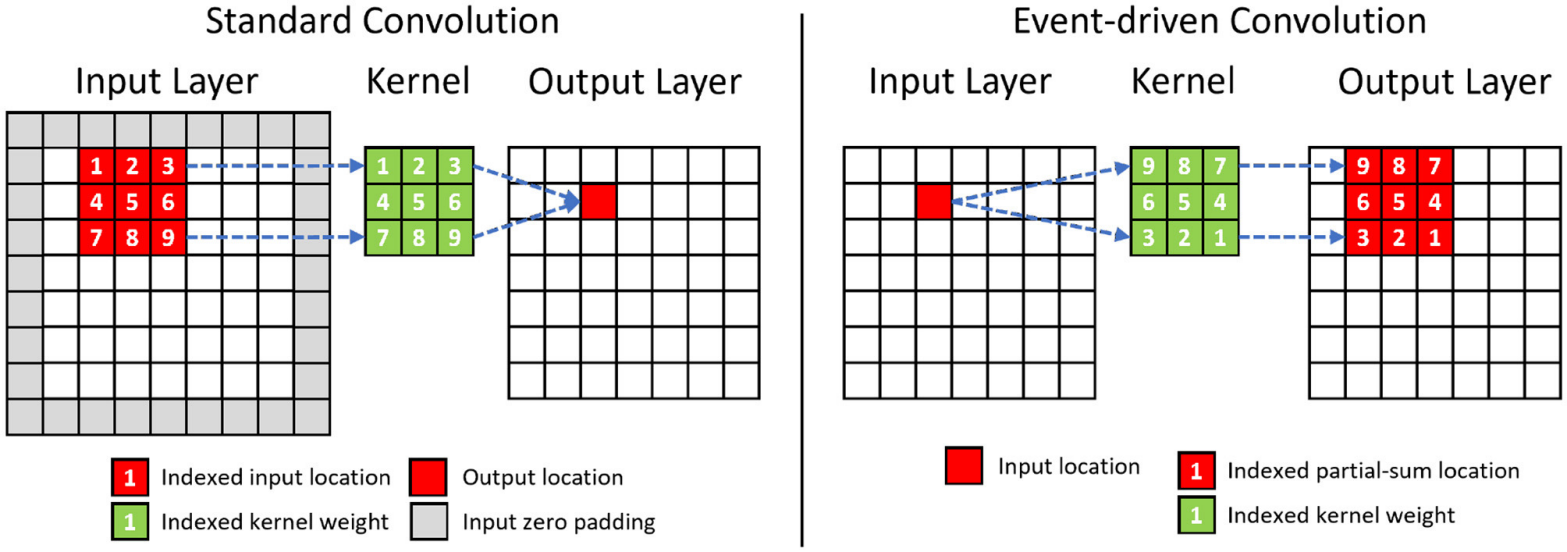
Comparison between the standard and the event-driven convolution. The event-driven convolution requires to rearrange the sequence of kernel weights. The change in the spatial sequence for a 3×3 convolution kernel is shown in the figure. The channel dimension of the tensor is omitted for simplicity.

Figure 3 shows the differences between the standard and event-driven convolution. The latter processes sparse events from the previous layer one by one in their order of arrival and accumulates them incrementally, directly into the neural states of the corresponding fanned-out postsynaptic neurons. However, this process requires maintaining high-dimensional neural states of convolutional layers in memory, which is impractical for the limited size of the on-chip memory, if the output tensor has a high dimension. To overcome this challenge, we propose the event-driven depth-first convolution.

**Figure 4:**
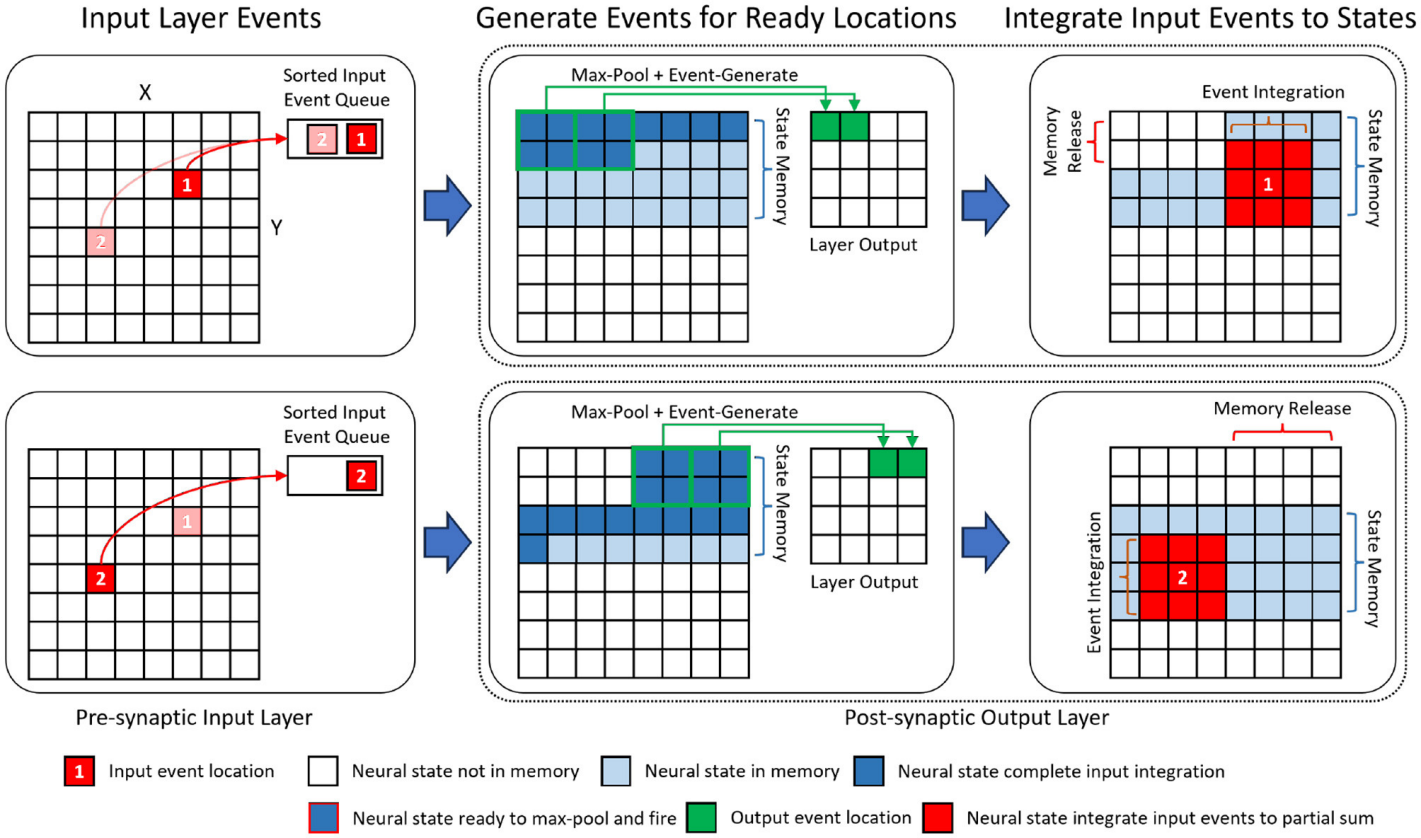
An illustration of the event-driven depth-first convolution on SENECA. We show a fused layer combining 3×3 convolution and 2×2 max-pooling. The layer processes input events sequentially from the sorted input event queue. Based on the location of the input, events are generated from spatial locations that have been fully updated, and their corresponding memory spaces are released. The channel dimension is omitted for simplicity.

Depth-first inference (Waeijen et al., 2021; Mei et al., 2023) is a scheduling method in neural network inference that prioritizes the network's layer (depth) dimension by consuming activations right after their generation. In our event-driven depth-first inference, the input events within a time step are assumed to be sorted in spatial order from the top-left corner of the (*X, Y*) plane to the bottom-right corner. Under this assumption, a neuron will receive all of its input events in a pre-defined order. Accordingly, its neural state updates will be concluded earlier than those of spatially lower-ranked neurons (Figure 4). As a result, it can fire immediately after its last neuron state update without needing to wait to process all the input events. After the event-generation process of a neuron, the memory for its neuron state can be released. As shown in Figure 4, for event-based depth-first convolution, each layer only needs to buffer a small portion of neural states that are incomplete/partially summed (the amount of required memory increases with the kernel size).

In event-based depth-first convolution, the cycle of **event-reception**, **neural processing**, and **event-transmission** is executed as a *tail-recursion* for each 2D coordinate (pixel location). Figure 4 illustrates the detailed procedure of our proposed event-driven depth-first convolution on a fused convolutional (kernel size 3×3, stride 1) and max-pooling (kernel size 2×2, stride 2) layer. We can divide this procedure into the following phases:

- When an event from the input location (*x, y*) has been received, all the neural states above the (*y*−1)*th* row or on the left of location (*x*−1, *y*−1) will not be updated further because there will not be any future incoming event that is within the kernel window view (3 × 3). Therefore, the event generation task will be triggered to generate the respective post-synaptic layer activations and then free up the memory storing the neural states.
- After firing the fully updated neurons, the input events at location (*x, y*) are processed. As a result, post-synaptic activity is generated at the same time as the input event trace is being processed. The event integration task integrates an input activation value to the neural states within the 3 × 3 spatial locations around the input location (*x, y*).
- If the neural states at a spatial location have been fully updated, the event generation task applies the activation function (e.g., ReLU) and 2 × 2 max-pooling function to the neural states to generate non-zero activation events. The event-sending function packs the non-zero activation events from the same spatial neuron location into an event stream with shared header information, including source neuron, number of events, etc. The event stream is then sent to the destination cores through the NoC.

As shown in Figure 4, the event-driven depth-first convolution requires storing (*K*+1) lines of neuron states per layer, equal to *X*×*C*×(*K*+1) neurons, where *X* is the spatial resolutions (height or width), *C* is the number of channels, and *K* is the width of the kernel. In Figure 4 where *K* = 3, neurons that are below the line (*X*+1) do not need to be stored because they have not received any spikes yet. Similarly, neurons that are above the line (*X*−2) also do not need to be stored because they have already fired and do not expect to receive any more spikes. Compared to other neuromorphic approaches that store all the neural states (*X*×*Y*×*C*) in the on-chip memory, the memory requirement for neuron states is significantly reduced by a factor of (*K*+1)/*Y*. Our efficient mapping strategy enables the mapping of a convolutional layer with a high spatial resolution to just one SENECA core.

Moreover, event-driven depth-first convolution can significantly reduce the inference latency when performing layer-to-layer event-driven data-flow processing in hardware. Traditional event-driven neuromorphic processing requires *barrier* synchronization at the end of the time step before event generation and communication. This introduces an additional latency per layer that equals the time required to integrate all the input events before a neuron can fire. The lock-step processing of Event Reception and Neural processing in depth-first convolution enables multilayered parallelism in a pipelined fashion across layers without the need for explicit per-timestep barrier synchronization primitives.

There are two main limitations of event-based depth first convolution. Firstly, this technique cannot be used in cases where recurrent and stateful neurons are required. This is because, for recurrent networks, the neuron states need to be kept alive to be used in the next time-steps. However, some studies have shown that it is not necessary to keep the states for all the convolutional layers in certain deep spiking convolutional neural networks (Perot et al., 2020). Therefore, for such layers, using the depth-first scheme can save a significant amount of memory. As a result, depth-first convolution can incentivize algorithm developers to optimize the efficiency of their network by using stateful neurons only where it is required.

As the second limitation, the proposed event-driven depth-first convolution requires the input events to be sorted and arrive in order. When using a conventional frame-based sensor (e.g. RGB camera), this requirement is automatically satisfied. However, an additional process is needed to sort the input events of the first layer when dealing with asynchronous events from event-based sensors. Nonetheless, the overhead is minimal if the input events are sparse.

### 2.4 Spike grouping

Despite the advantages of event-driven processing, it imposes significant per-spike overheads during **event-reception** (unpacking each event and preparing the task) and **neural processing** (read/write neuron states per each event). The time and resources required for these steps can easily dominate the overall costs. For example, as shown in Tang et al. (2023a), a single memory access for the data movement from SRAM to registers can be more than twice the cost of an arithmetic instruction.

As mentioned, processing each spike requires the following steps: (1) event decoding or projecting the spike address to several physical addresses of the weights and neuron states in data memory, (2) reading the relevant weights and neuron states from the data memory, (3) performing the neural calculation, and (4) writing the updated neuron states to the data memory.

To reduce the processing cost per event, we suggest grouping spikes with specific constraints and treating them as a single event. For instance, in this study, spikes that occur in the same time step and target the exact same neurons in the next layer are combined as a group. Packing such spikes that share the same destination neuron addresses significantly reduces the overhead of event decoding (step 1). Then, during the neural processing step, a neuron's state can be read once and updated multiple times by the grouped events before it is stored back into memory, considerably reducing memory accesses (steps 2 and 4).

**Figure 5:**
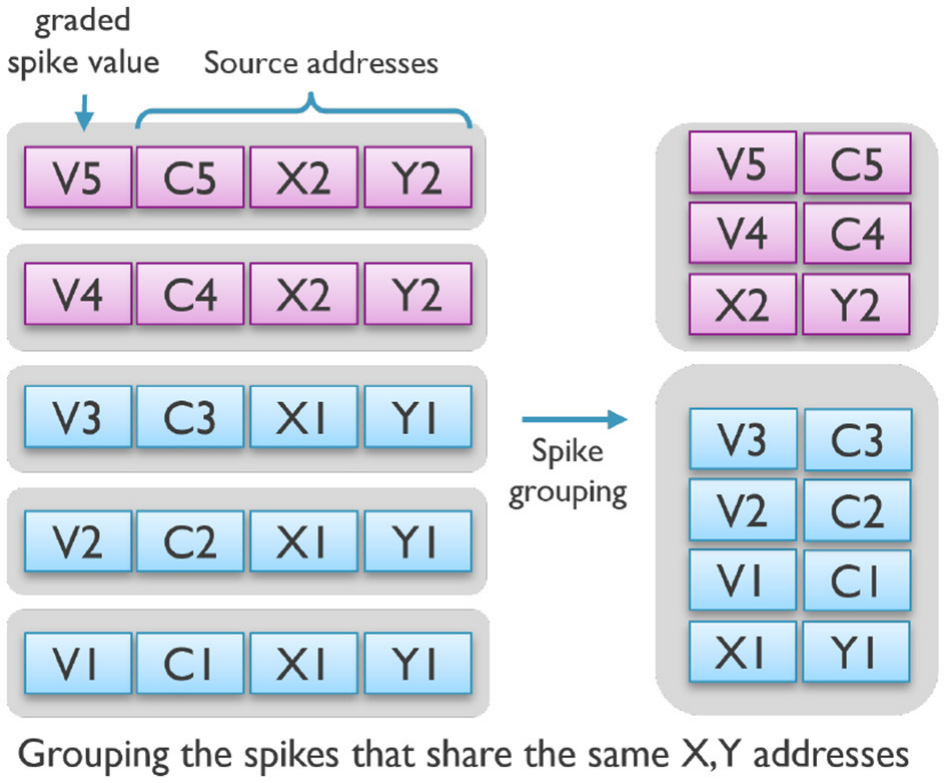
The spike-grouping technique groups together spikes with similar source addresses, reducing communication, and memory access.

Furthermore, as shown in Figure 5, since these events share part of their spike address, grouping them together decreases the communication overhead by reducing the number of overall address bits per spike in a spike-group event and minimizing data movements between cores.

As we discuss later in our results, spike grouping with a maximum of four spikes in one group reduced the average energy/latency cost of synaptic operation on SENECA by half.

### 2.5 Hardware-aware training

Hardware-aware training is required to fully exploit the benefits of event-driven computation on processing latency and energy efficiency. Our hardware-aware training produces a model with sparse activation patterns and low-precision parameters, which will reduce synaptic operations and data movements on SENECA.

To improve the efficiency of neural networks, we use sparsity-aware training which penalizes positive neural states and replaces the ReLU activation function with FATReLU (Forced Activation Threshold ReLU) (Kurtz et al., 2020). FATReLU uses a trainable activation threshold. Additionally, we use quantization to reduce weight precision to 4*b* integer, with a shared power-of-two scaling factor for all the weights of the same layer. Other network parameters are quantized to 16-bit BrainFloat.

To maintain high model accuracy, we adopt an incremental quantization-aware training strategy. We start by quantizing the parameters of the first layer after training for *N* epochs. Then we freeze the first-layer parameters and proceed to quantize and freeze the second-layer parameters after 2*N* epochs, and so on for all layers.

## 3 Experiments and results

To demonstrate the advantages of our optimized event-driven computing paradigm, we performed experiments on mapping different neural networks on the SENECA neuromorphic architecture. This section comprises three major components. First, we demonstrate the exploration process we conducted to optimize the event-driven computing on SENECA via hardware-algorithm co-optimization. Specifically, we show step-by-step how to achieve optimal event-based neural network inference with the proposed spike-grouping processing for the keyword spotting task. Second, we benchmark our event-driven depth-first convolution on visual recognition tasks. We compare the benchmarking results with state-of-the-art event-driven neuromorphic and conventional solutions. Third, we trained a larger neural network using the challenging Prophesee 1M Pixel automotive detection dataset (Gen4) (Perot et al., 2020) and presented in-depth benchmarking results. As far as we know, this is the first paper to report the results for this task on a neuromorphic processing platform. Therefore, we were unable to compare our results with others. Overall, the experiments validate our optimization techniques on the event-driven paradigm and show the advantages of our proposed event-driven depth-first convolution regarding energy, latency, and area efficiency.

### 3.1 Hardware measurement setup

All hardware-related measurements were performed in gate-level simulation (post-synthesis) using industry-standard ASIC simulation and power measurement tools (Cadence Xcelium and Cadence JOULES) for GF-22*nm* FDX technology node (in the typical corner 0.8*V* and 25*C*, no back-biasing, 500MHz clock frequency). Even though the physical layout (place and route) is not done, the design flow still estimates the parasitic effects of wires in the actual IC. Therefore, the power results are accurate within 15% of signoff power and include the total power consumption of the chip, i.e. both dynamic and static power. We have not included the I/O power consumption in the reported results. In the reference comparison with other chips, they may report either the total power (static and dynamic) or only the dynamic power of the core [e.g., Loihi (Blouw et al., 2019)].

### 3.2 Step-by-step architectural benchmarking on keyword spotting task

To evaluate the effects of the various design choices in the SENECA architecture, we perform an ablation study that reveals the contribution of each component in the architecture to the total latency and energy consumption of the system. In order to be able to compare with other neuromorphic processor architectures (Blouw et al., 2019; Yan et al., 2021), we mapped the “ABR Keyword Spotting (KWS) Power Benchmarks” (Blouw et al., 2019) in one SENECA core, using a similar fully connected neural network. The KWS task processes an audio stream to detect some keywords of interest (e.g., "Aloha"). The ablation study is carried out using a sequence of step-by-step experiments.

#### 3.2.1 Algorithm

We deployed the same 3-layer fully connected feed-forward neural network used in Blouw et al. (2019), which has 390 input neurons, two dense layers with 256 neurons each, and an output layer with 29 outputs. The input of the network is a 390-dimensional Mel-frequency cepstral coefficient (MFCC) feature of the audio waveform. The network's output is a 29-dimensional representation of English characters together with special characters such as silence. During inference, ten time-steps of MFCC features are fed to the network, and the final results are obtained by post-processing the generated outputs. For the model instantiated on SENECA, all synaptic weights were quantized to 4b by the post-training quantization process^1^, which reduced the test accuracy by 2% (from 95% to 93%). We only quantized the weights while keeping the bias parameters and neural states at higher precision.

#### 3.2.2 Step-by-step experiments

**Figure 6:**
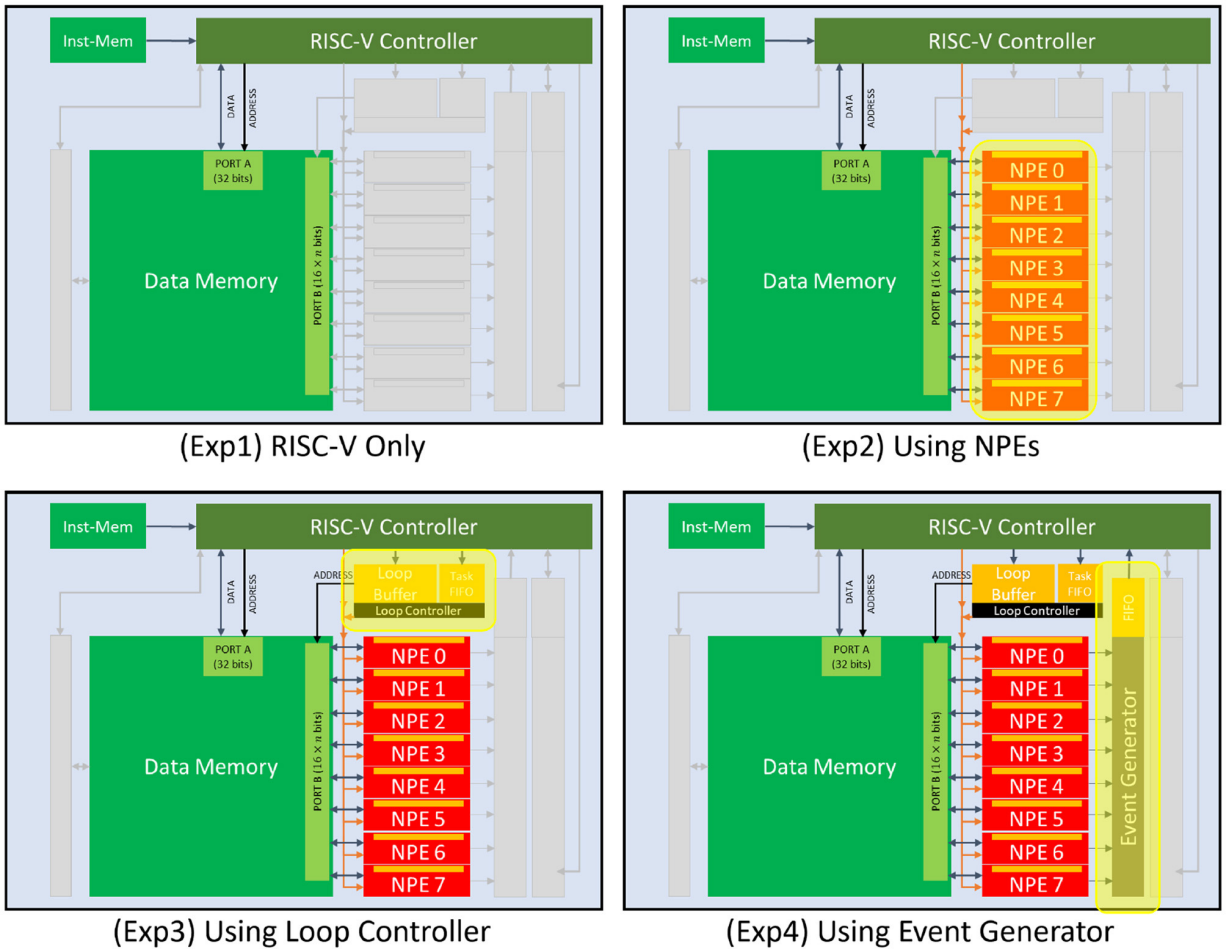
Accelerators used in the KWS experiments. Experiment 5 (Spike-grouping) is a pure mapping optimization that uses all the accelerators (same as Experiment 4).

To quantify the increase in efficiency brought by each of the architectural blocks in the architecture, we start with a baseline experiment where the entire data path of the neural computation (model inference) takes place inside the RISC-V controller. Then we introduce additional experiments where we incrementally enable components of the architecture to accelerate parts of the data path computation and measure its effect (Figure 6):

- Experiment 1 (**RISC-V only**): We used the RISC-V controller of the SENECA core to perform the neural network computations. RISC-V controller can only perform integer (fixed-point) operations. In this experiment, the synaptic weights are 4b integer values. Biases and neuron states are int32, the native precision of the RISC-V (IBEX) (Schiavone et al., 2017; Chadwick, 2018).
- Experiment 2 (**+** **NPEs**): We offload the neural computations to NPEs. In this experiment, the synaptic weights are similarly 4b; however, biases and neuron states are in Brain Float (16b), the native precision of the NPEs. The RISC-V is responsible for event preprocessing, address calculations for NPE operations, and event post-processing (packetization).
- Experiment 3 (**+** **Loop controller**): We use the loop controller to take over (from the RISC-V) the acceleration of address calculations and orchestrate the time-multiplexing of repetitively executed SIMD microcodes to the NPEs (for various neural computation tasks).
- Experiment 4 (**+** **Event generator**): We add the event generator accelerator to the data path to take over the task of converting the neuron activations (spikes) to AER events.
- Experiment 5 (**+** **Spike-grouping**): This experiment has added the optimizations for spike-grouping to reduce total memory accesses required in the data computations. This allows the reuse of loaded neuron states inside the NPEs several times before storing them back in the data memory.

The results of these experiments are summarized in Table 1. One can see that RISC-V alone consumes a lot of energy and time per inference. Using NPEs in Experiment 2 to perform neural operations improves the inference time by 6× and reduces the total energy consumption by 5× from Experiment 1. Involving the loop controller (Experiment 3) further improves the inference time by 2× and reduces the total energy consumption by 2.4×. Along the same lines, Experiment 4 shows that the event generator improves the inference time by 1.3× and reduces the total energy consumption by 1.3× ; and Experiment 5 shows that spike-grouping improves the inference time by 1.8× and reduces the total energy consumption by 1.7×. Overall, with all optimizations (Experiment 5), the inference time is improved by 30× and the total energy consumption is reduced by 28×, compared to the RISC-V-only implementation (Experiment 1).

**Table 1:** Results comparison for various implementations of the KWS task on SENECA.

**Experiment**	**Inference**	**RISC-V**	**NPEs**	**Dmem**	**Total Core**
	**Time (*μS*)**	**Energy (*μJ*)**	**Energy (*μJ*)**	**Energy (*μJ*)**	**Energy (*μJ*)**
(1) RISC-V only	6,625	29.1	0.013	1.67	34.0
(2) +NPEs	1,098	2.87	1.91	1.45	6.71
(3) +Loop Controller	541	0.23	1.29	1.16	2.81
(4) +Event Generator	400	0.24	0.92	0.83	2.10
(5) +Spike-grouping	218	0.17	0.50	0.47	1.20

All reported time and energies are for one inference (averaged over the dataset). RISC-V energy includes RISC-V and its instruction memory. NPEs energy includes all NPEs, Loop buffer and Event-generator blocks. In all the experiments, the accuracy is the same (93%). The reported energy numbers include the leakage power of 30μ*W*.

#### 3.2.3 Comparison with other platforms

We compare SENECA against various other neuromorphic processors on the KWS task in terms of accuracy, energy and latency (Table 2). The KWS benchmarking task is initially introduced to benchmark intel Loihi (Blouw et al., 2019). Loihi's measurement results show 10× higher energy consumption than our RISC-V-only implementation (Experiment 1) of SENECA. We believe the main reason for this inefficiency is the use of rate coding in the model of the KWS neural network. Rate coding converts every neuron activation into a train of spikes just to communicate a single graded (non-binary) value.

**Table 2:** Results comparison for KWS deployment in various hardware architectures.

**Hardware**	**Accuracy (%)**	**Inference per second**	**Energy/ inference (*μJ*)**
Loihi (Blouw et al., 2019)	93.8%	296	372
SpiNNaker2 (Yan et al., 2021)	93.8%	1,000	7.1
SENECA	93%	4,587	1.2

SpiNNaker2 has a very similar architecture to SENECA, using a MAC array next to a ARM processor. It is also based on the same technology-node as SENECA (GF-22nm FDX). SpiNNaker2 performs better than Loihi regarding inference time (3.3× ) and energy (52× ). We believe the main reason for this better performance is the flexibility of SpiNNaker2 architecture, which allows the implementation of graded spikes. Using graded spikes removes the requirement of rate-coding in the model implementation. Graded spikes optimization is also supported by the new version of the Intel Loihi chip (Orchard et al., 2021) (however, benchmark results of Loihi2 are not available yet). The main difference between SpiNNaker2 and SENECA is the lack of a hierarchical task-controlling system (RISC-V and loop controller). Therefore, the SpiNNaker2 results are similar to SENECA results in Experiment 2 of Table 1, where we use NPEs without the loop controller.

### 3.3 Event-driven depth-first convolution for visual recognition and sensor fusion

The proposed event-driven depth-first convolution promises to increase computation and memory efficiency in multi-core neuromorphic processors. To characterize and quantify the improvements, we carry out experiments in two classification tasks: gesture recognition (Ceolini et al., 2020) and handwritten digit classification (Deng, 2012). The selection of tasks was made to allow benchmarking against other neuromorphic processors. We used the same convolutional neural network (CNN) for both experiments, mapped on four SENECA cores.

The gesture recognition task (Ceolini et al., 2020) requires fusing signals from electromyography (EMG) and vision sensors in order to identify hand gestures. EMG signals are captured using electrodes on the subject's wrist to measure electrical signals produced by muscle activity. In this dataset, two vision sensors are used to capture the subject's hand spatial motion: 1) Dynamic Vision Sensor (DVS), which generates spikes in response to changes in light intensity, and 2) Active Vision Sensor (APS), a gray-scale camera with 240 × 180 pixels resolution. The dataset comprises five sign language gestures. The visual and EMG signals are synchronized. There are 63 recording sessions from 21 subjects, over 15k samples in total. The DVS was attached to a random moving platform during the recordings to capture information similar to that captured by the APS vision sensor.

The handwritten digit classification task uses the MNIST dataset (Deng, 2012), which allows us to benchmark our platform against a wider range of acceleration options. The dataset consists of 60,000 training and 10,000 testing images with 28 × 28 pixels resolution. Some neuromorphic processors reported their results on the N-MNIST dataset (Orchard et al., 2015). It is an equivalent dataset that converts the MNIST dataset to spikes using a DVS camera.

#### 3.3.1 Algorithm

**Figure 7:**
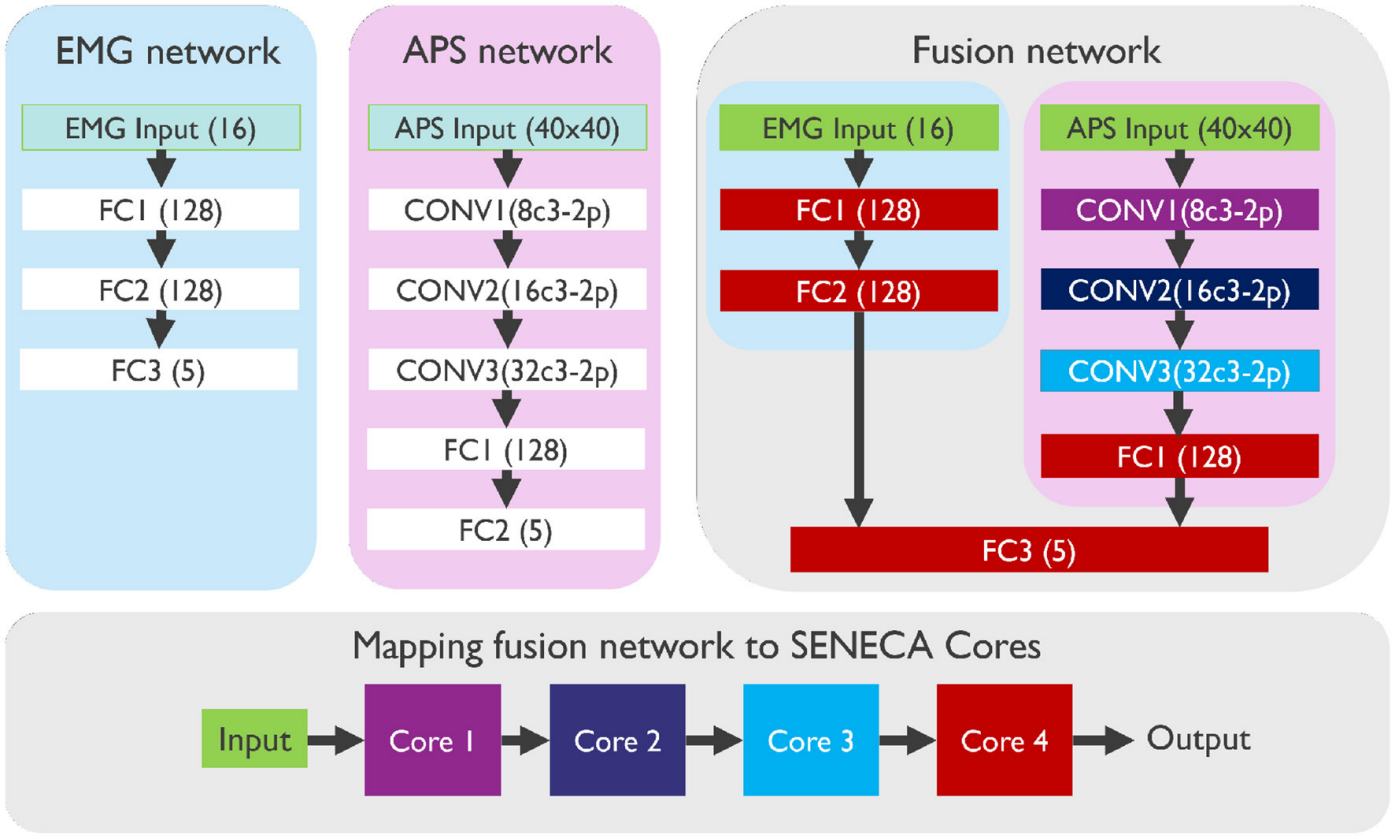
Architectures of EMG, APS, and Fusion neural networks, mapped on four cores of the SENECA neuromorphic platform. The color of each layer in the fusion network corresponds to the core where it is mapped.

While some Neuromorphic processors (Lines et al., 2018) only process binary spikes as inputs or neuron activations, which necessitates a pre-processing step to convert multi-bit input data (e.g. measurement of the EMG sensor), SENECA, on the other hand, is capable of handling graded spikes. So, we did not need to convert the analog EMG sensor measurements into graded spikes directly. Additionally, we found that the APS vision sensor is more suitable for our event-driven depth-first inference due to the potential of the APS sensor to directly output naturally sorted pixel values. To process APS data, we convert each pixel value to a graded spike and drop the values that are smaller than a specific threshold. We used three neural network architectures: two for processing independently EMG data and APS data and one to perform sensor fusion with both modalities, as illustrated in Figure 7:

- The **EMG network** contains three fully connected (FC) layers with Input(16)-FC1(128)-FC2(128)-Output(5) neurons.
- The **APS network** contains three convolutional layers (CONV) and two fully connected layers (FC): Input(40 × 40× 1)-CONV1(8c3-2p, 20 × 20× 8)-CONV2(16c3-2p, 10 × 10× 16)-CONV3(32c3-2p, 5 × 5× 32)-FC1(128)-Output(5). XcY denotes a convolution layer with X kernels of shape Y-by-Y, while 2p denotes a 2-by-2 max pooling. All CONV layers have stride 1 for convolution and stride 2 for max pooling.
- The **Fusion network** contains both the APS and the EMG networks. The last fully connected layers of both, are then merged into one fully connected layer.

Our neural network architecture is based on the baseline from Ceolini et al. (2020), albeit with smaller FC1 layers for the APS network and the fusion network. All layers (except from the output layers) use FATReLU (Forced Activation Threshold ReLU) (Kurtz et al., 2020) as the activation function. As a result, the overall MAC operation sparsities of the trained EMG, APS, and fusion networks are 79%, 88%, and 88%, respectively.

For the handwritten digit classification task, we used the same network architecture and training method as the APS network in the gesture recognition task. The MNIST image frames have lower resolution (28 × 28), which results in less memory required for neuron states. The overall operation sparsity (assuming no weight sparsity) of the trained digit classification network is 80%.

#### 3.3.2 Gesture recognition benchmark results

Table 3 presents the model and performance metrics of the gesture recognition task on SENECA and a comparison with a few other hardware accelerator platforms from the literature. Our implementation outperforms others in accuracy, energy, and latency aspects.

**Table 3:** Results comparison for gesture recognition task with a single sensor and sensor fusion in various hardware architectures.

**System**	**Modality**	**Accuracy**	**Inference**	**Inference**	**Silicon**	**Number**
		**(%)**	**Energy (*μJ*)**	**Time (*ms*)**	**Area (*mm*^2^)**	**of cores**
Spiking CNN	EMG	55.7	173.2	5.89	2.4	6
(LOIHI)	DVS	92.1	815.3	6.64	39	95
(Ceolini et al., 2020)	Fusion	96.0	1104.5	7.75	41	100
Spiking MLP	EMG	53.6	7.42	23.5	0.08	1
(ODIN +MorphIC)	DVS	85.1	57.2	17.3	2.86	4
(Ceolini et al., 2020)	Fusion	89.4	37.4	19.5	2.9	5
Event-driven	EMG	67.34	0.147	0.019	0.47	1
CNN(SENECA)	APS	94.75	16.9	2.15	1.88	4
	Fusion	96.52	17.6	2.16	1.88	4

The Silicon area is the total area of the utilized cores. The memory capacities and technology nodes of each core are: Loihi = 2 Mb in 14 nm, ODIN = 286kb in 28 nm, MorphIC = 576 Kb in 65 nm, and SENECA is 2.3 Mb in 22 nm.

Unlike other neuromorphic chips, SENECA achieves faster inference due to the single-step and depth-first convolutional processing using graded spikes. For instance, Loihi requires 200 time-steps for one inference in the fusion network, whereas SENECA only needs one time-step.

**Figure 8:**
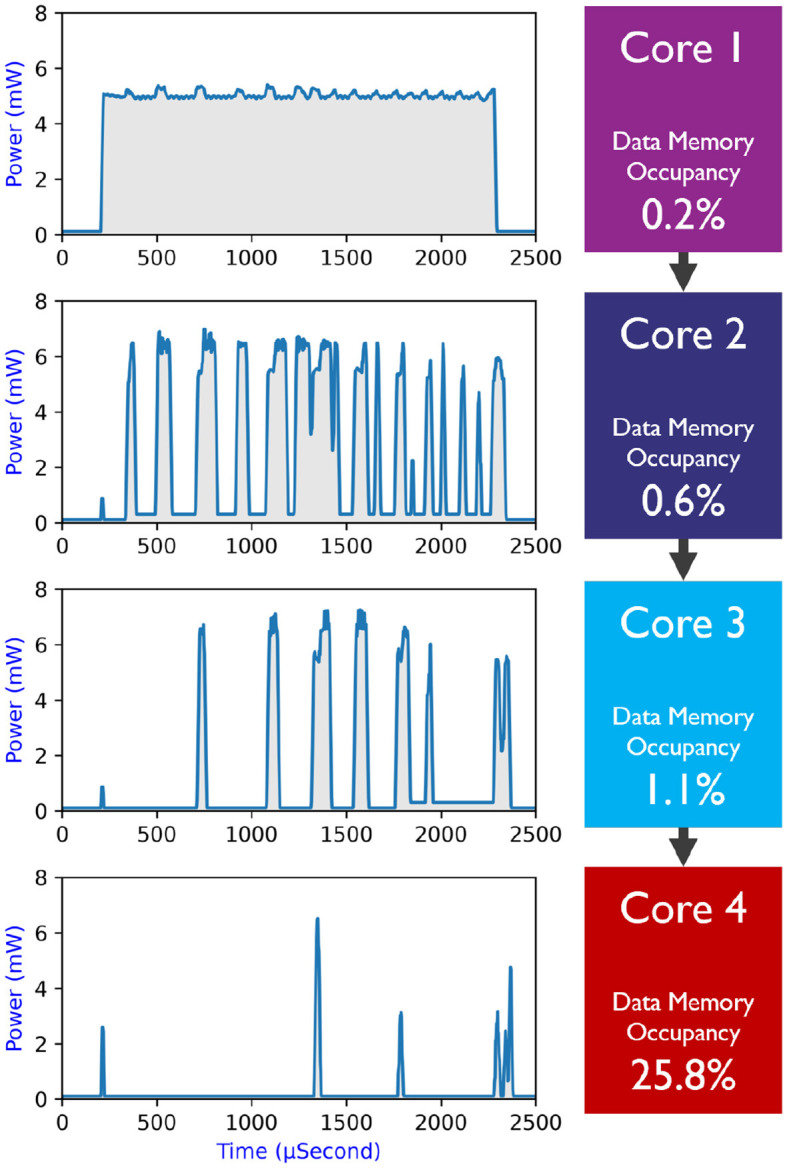
SENECA cores' resource utilization for fusion network. **(Left)** Power consumption of each core in time, and **(right)** the data memory utilization of each core. The total data memory size is 2 Mb (256 KB). The current mapping is straightforward but not fully optimized for best performance and area efficiency.

The Silicon Area in Table 3 is extracted by adding up the area of the used cores in the neuromorphic platforms. In contrast to the one hundred Loihi cores used in a previous study (Ceolini et al., 2020), we could map the fusion network into only four SENECA cores as shown in Figure 7, thanks to the proposed memory-efficient mapping technique (event-driven depth-first convolution). Considering that SENECA and Loihi have similar memory capacities per core, our approach achieved a remarkable 25× improvement in silicon area efficiency. However, as shown in Figure 8, there is still room for improvement since the resource utilization is unevenly distributed among the SENECA cores. Therefore, there is potential to increase the area efficiency with further optimization efforts. For instance, multiple CONV layers can be fused together in a single core. Additionally, when it comes to FC layers, they have less computing but more memory requirements, as shown in Figure 8. In this case, utilizing shared memory (slower but denser) can prove to be beneficial.

#### 3.3.3 MNIST benchmark results

Analogous results and comparisons for the MNIST handwritten digit classification task on SENECA are reported in Table 4. Our implementation outperforms other hardware platforms regarding accuracy, energy consumption, and latency. The second fastest deployment is reported in IBM TrueNorth (Esser et al., 2015), which performs a single-step MNIST processing using binary weights and activations. However, the TrueNorth implementation requires 1,920 cores and consumes 192Mb of memory (compared to 4 cores and 8Mb memory in SENECA). On the other hand, SENECA is four times faster, mainly due to depth-first convolutional processing. Overall, SENECA consumes more than 9× less energy due to the significantly more memory-efficient mapping that results in efficient deployment in only four cores.

**Table 4:** Results comparison for MNIST handwritten digit classification task in various hardware architectures.

**System**	**Dataset**	**Accuracy**	**Inference**	**Inference**	**Silicon**
		**(%)**	**Energy (*μJ*)**	**Time (*ms*)**	**Area (*mm*^2^)**
Event-Driven CNN	MNIST	99.44	12	1.1	1.88
(SENECA)					
Spiking CNN (LOIHI)	MNIST	99.21	660	6.65	5.74
(Rueckauer et al., 2022)	N-MNIST	98.51	620	7.07	12.3
Spiking CNN (Speck)	N-MNIST	98.56	180	300	30
(Richter et al., 2023)					
Spiking CNN (TrueNorth)	MNIST	99.42	108	4	192
(Esser et al., 2015)					
CNN (MAX78000)	MNIST	99.44	215	8.3	NA
(Moss et al., 2022)					

The Silicon area is the total area of the utilized cores.

Despite not being a neuromorphic chip, ADI's MAX78000 in Table 4 shows competitive performance to other neuromorphic platforms, due to its efficient usage of on-chip memory. In comparison to SENECA, MAX78000 does not exploit activation sparsity during inference. This means the amount of activation sparsity does not affect the performance of MAX78000. To highlight the impact of activation sparsity on SENECA, we deployed a model of the neural network that generates denser activations (50% sparsity compared to the previously reported 80%, produced without activation-sparsity-aware training) and measured its performance. Execution of this less compute-optimal network model in SENECA increased the average inference energy by 2× and the average inference latency by 1.5×.

### 3.4 In-depth benchmarking for high-resolution automotive object detection task

In this section, we are presenting a detailed report on the performance of different layers and accelerators in SENECA for a larger neural network. Through this benchmarking, we aim to gain valuable insights into the hardware architecture, sparsity, and layer dimensions' impact on the system's performance. To achieve this, we chose one of the most challenging neuromorphic datasets available, the Prophesee 1M Pixel automotive detection dataset (Gen4) (Perot et al., 2020). Our event-driven depth-first inference approach enables us to process high-resolution visual inputs without excessive use of the on-chip memory. Although other neuromorphic platforms have benchmarked on smaller object detection tasks (Caccavella et al., 2023), we are the first neuromorphic systems that have benchmarked with this large-scale dataset. Therefore, in this section, we have provided an in-depth benchmarking report of SENECA without any comparison to other systems.

**Figure 9:**
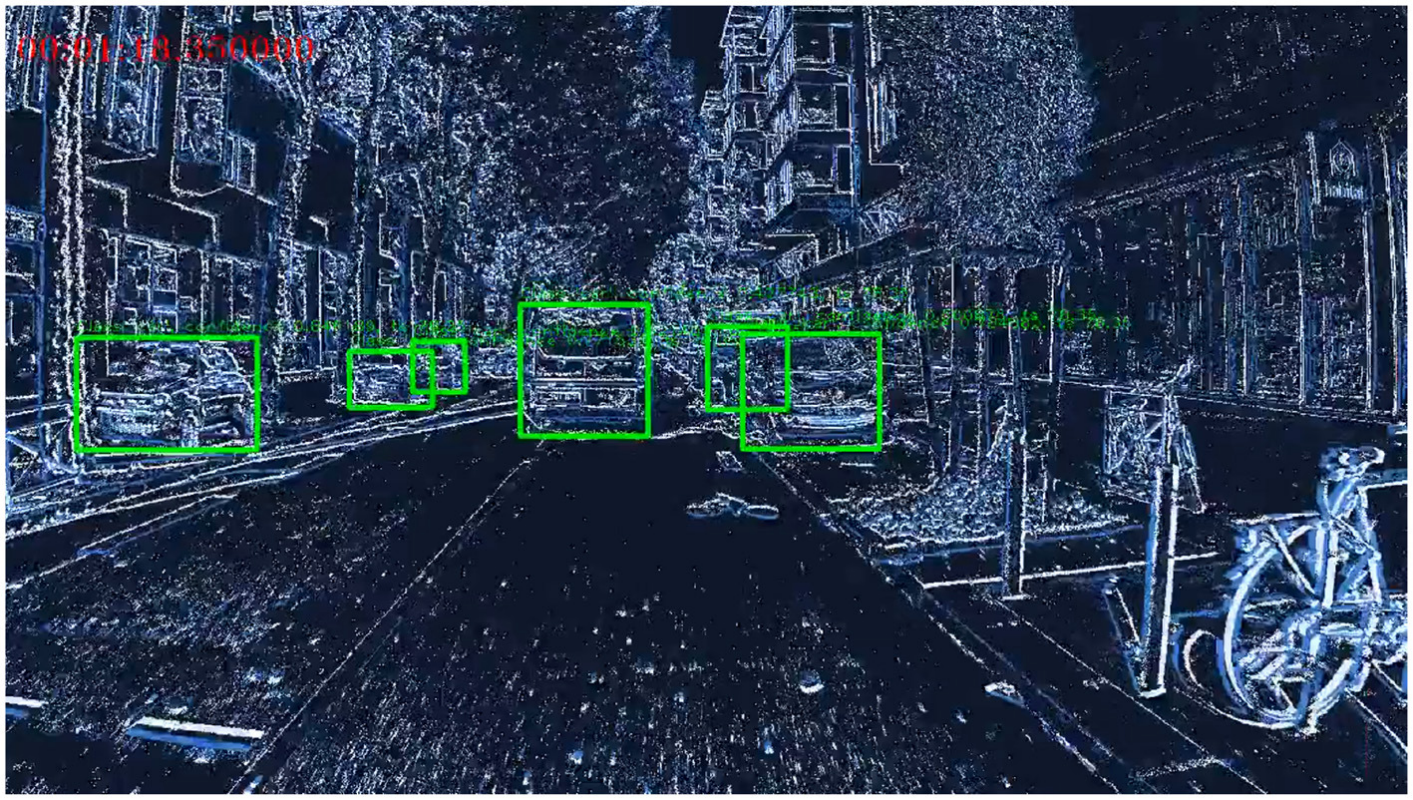
A snapshots of the Gen4 dataset, along with its corresponding labels.

The Prophesee 1M Pixel automotive detection dataset (Gen4) (Perot et al., 2020) has been recorded by placing a 1MP event-based camera (1280 × 720) in front of a car. The dataset contains 15 hours of street view recording and includes 25M bounding boxes around six different object categories. However, only three classes of objects are used for benchmarking: Cars, two-wheels, and Pedestrians. Figure 9 shows a snapshot of the recordings in this dataset.

#### 3.4.1 Algorithm

**Figure 10:**
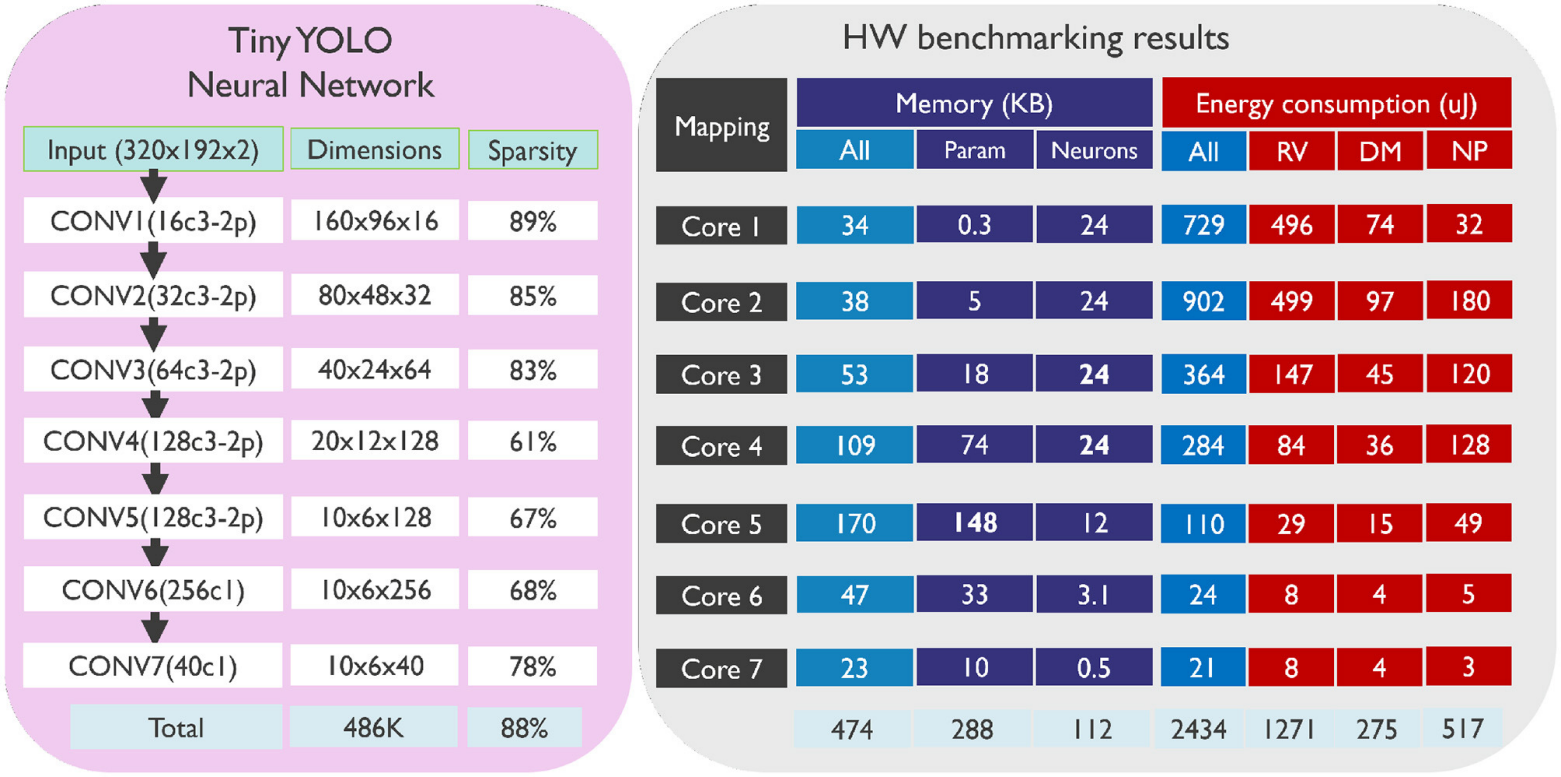
Tiny YOLO CNN architecture used for the object detection task (Gen4 dataset), along with the details on how the mapping has been done on SENECA cores and information about memory and energy consumption. The memory usage per core includes the total occupancy of the data memory (instruction memory consumption is about 20KB for all cores). Additionally, memory used for neuron states (16b per neuron) and parameters (weights and biases, 8b) are reported separately. The energy consumption is averaged over the entire dataset to infer a 50ms event stream and is presented in four columns: “All” shows the total energy consumption of the core, while “RV” is RISC-V and its instruction memory, “DM” is Data Memory, and “NP” is all the Neural Processing accelerators: NPEs, loop controller and event generator.

Figure 10 shows the seven-layer tiny YOLO CNN structure that we used for object detection in this task (XcY denotes a convolution layer with X kernels of shape Y-by-Y, while 2p denotes a 2-by-2 max pooling). All CONV layers have stride 1 for convolution and stride 2 for max pooling. We also included the dimensions for each layer, which indicate the number of neurons in each layer. The input is down-sampled to 320 × 192 since higher resolution input did not improve the network accuracy. This CNN has 486K neurons and 288K parameters.

**Figure 11:**
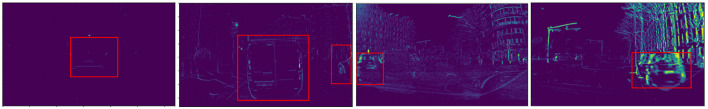
Prediction of our tiny YOLO CNN for four different scenes in the dataset with varying levels of sparsity (99.9%, 98%, 89%, 77%).

We used Prophesee meta-vision libraries to convert input events into histogram data points. To increase sparsity, we added a fixed threshold during both the training and inference phases. Figure 11 displays four scenes of the dataset using the preprocessing technique with varied levels of sparsity and also depicts the predictions of our SENECA platform.

The last layer of the network has a total of 5× (3+4+1) channels^2^. To achieve a COCO mean Average Precision (mAP) of 16, we quantized all parameters to 8 bits while still using the 16b floating point (BF16) format for neuron states. The average amount of operation sparsity in this network is 88%. We used the same technique as described in Section 3.3.1 for sparsification and quantization.

#### 3.4.2 Detailed benchmarking results

Figure 10 illustrates the detailed results of benchmarking our tiny YOLO network for the Gen4 dataset. Same as before, we decided to use a straightforward mapping of one layer per core. The memory consumption of each core includes the parameters, neuron states and other overheads (such as stack). The depth-first inference method introduced in this work consumes 8 times less neuron state memory (112 KB for 486K neurons) than a conventional solution.

In Figure 10, we can see the energy consumption for each layer/core. The total energy consumption for inferring a single data point (50 ms events) is 2.4 mJ. The figure also shows the energy distribution between RISC-V, data memory, and neural processing accelerators. As mentioned earlier, RISC-V performs address calculation for each input event at every (X, Y) location. Therefore, RISC-V's energy consumption depends on the (X, Y) dimensions and sparsity but not on the Channel dimension. For the layers with lower (X, Y) dimensions and higher sparsity, RISC-V's energy consumption is reduced. On the other hand, the energy consumption of neural processing accelerators and data memory depends on all layer dimensions (X, Y, C) and sparsity. Neural processing accelerators perform all the important computations, but the energy consumption of RISC-V is still high. This is because the depth-first inference method needs a complex address calculation process, which consumes more energy. To address this issue, further research is needed to create a specialized accelerator that can assist RISC-V with depth-first address calculation.

**Figure 12:**
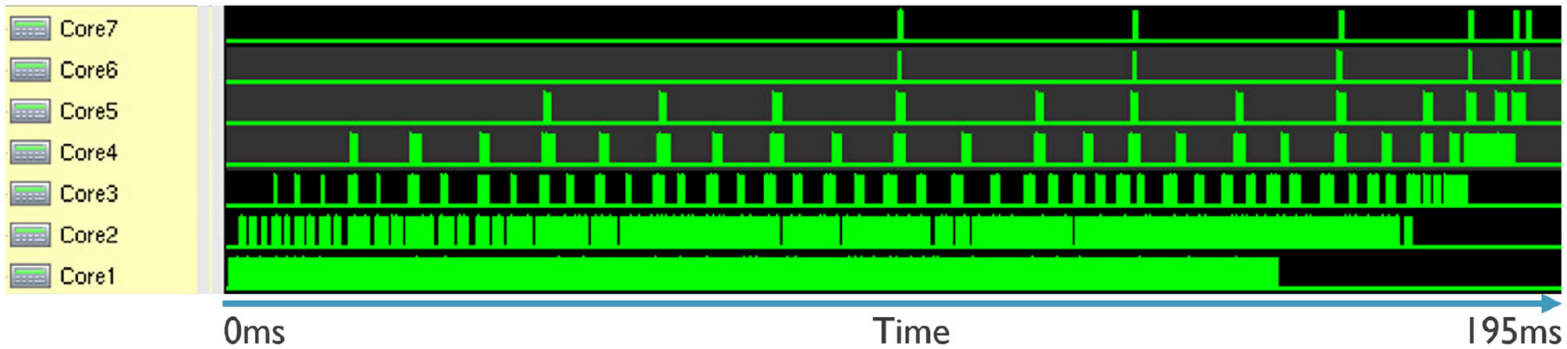
Snapshot of activity of each core in time for the inference of one data point (50 ms of events) with the average sparsity.

Figure 12 displays the activity of each core over time. It shows that, for the current mapping of our application, it takes 195 ms to process 50ms of events. This means that the processing is about four times slower than real-time. However, we can see that the mapping is not optimized since only the first two cores are busy, while other cores have very little to do. By redistributing the layers or performing hardware-aware training, it is possible to improve the latency.

It is important to note that the measurement was taken for a data point with an average amount of sparsity. Therefore, the latency can be better or worse, depending on the input data. Additionally, the graph shows the scheduling of depth-first inference. This allows the next core to start processing before the previous core has finished. This depth-first scheduling improves the latency of the system, as can be seen in the graph.

## 4 Discussion and limitations

This paper explored optimizations of neural network processing on an event-driven neuromorphic accelerator with the aim of improving latency, energy, and silicon area efficiency. Spike-grouping alleviates the high overheads of per-event processing, and event-driven depth-first convolution improves the mapping efficiency in digital neuromorphic accelerators. The flexibility of the SENECA architecture allowed the exploration and characterization of the proposed optimization techniques. These optimizations improve the state-of-the-art hardware performance of event-based neural network processing on digital CMOS accelerators.

While we did not assess the power consumption of certain critical units, such as I/Os (because of resorting to hardware simulation tools), nevertheless, our results are still significantly better than the current state-of-the-art, even when using pessimistic estimates. On the positive side, using simulated power measurement platforms allowed us to perform detailed sub-system power breakdown measurements as reported in this paper.

It is worth mentioning that although the proposed event-driven depth-first convolution addresses the problem of memory cost, there are side issues to be considered. Namely, the processing requirement to spatially sort input events. This may add pre-processing overhead for asynchronous neuromorphic sensors, such as event-based cameras (Gallego et al., 2020).

In addition, if a layer is to be mapped to multiple asynchronously operating cores, its output to the next layer needs special care since the input events to the next layer may arrive out of order.

Despite the limitations, the presented optimizations complement recent advancements in neuromorphic algorithm designs (Yik et al., 2023). The cost of event-driven depth-first convolution can be further reduced by adopting cutting-edge activation-sparsity-aware training methods (Kurtz et al., 2020; Zhu et al., 2023). This can gain even more significant improvements in real-world applications requiring more complex and deeper neural networks, which are due to be explored in the coming works. Additionally, our optimizations promise to reduce the latency of stateful convolutional networks by increasing parallelism and decreasing event overheads. Developments in hybrid networks combining non-stateful and stateful convolutional layers present another possibility for optimizing neuromorphic solutions that take advantage of both sides. With a similar hardware-algorithm co-optimization strategy, future event-based neural network algorithms (Schuman et al., 2022), for instance, synaptic delay (Patiño-Saucedo et al., 2023) and learning (Tang et al., 2021), can be further optimized for neuromorphic processors.

We showed that a flexible digital neuromorphic processor can result in better hardware performance than inflexible designs. This contradicts the traditional thinking that flexibility always comes with a cost of efficiency (Garcia et al., 2006). In the case of digital neuromorphic processors, the inflexible design largely limits the optimization space for event-based neural network processing. Our results demonstrated that the benefits of having a large optimization space to explore outweigh the cost of enabling flexibility (in a strategically designed architecture). Nevertheless, the proposed event-driven depth-first convolution can result in a specialized hardware design, further increasing the performance in real-world applications by reducing the control overheads.

## Data availability statement

The original contributions presented in the study are included in the article/supplementary material, further inquiries can be directed to the corresponding author.

## Author contributions

YX: Investigation, Methodology, Software, Validation, Writing – original draft, Visualization, Conceptualization. KS: Investigation, Methodology, Software, Validation, Writing – original draft, Visualization, Conceptualization. G-JS: Investigation, Methodology, Software, Writing – original draft, Visualization, Funding acquisition, Supervision, Conceptualization, Project administration. RB: Software, Validation, Writing – review & editing. AD: Software, Validation, Writing – review & editing. SW: Software, Validation, Writing – review & editing. RM: Software, Validation, Writing – review & editing. PN: Software, Validation, Writing – review & editing. CA: Software, Validation, Writing – review & editing. PM: Software, Validation, Writing – review & editing. AG: Supervision, Methodology, Writing – review & editing. SH: Supervision, Methodology, Writing – review & editing. PD: Investigation, Methodology, Software, Writing – original draft, Visualization, Funding acquisition, Supervision, Conceptualization, Project administration. ST: Project administration, Methodology, Writing – review & editing. MK: Project administration, Methodology, Writing – review & editing. KV: Investigation, Methodology, Software, Writing – original draft, Visualization, Funding acquisition, Supervision, Conceptualization, Project administration. MS: Investigation, Methodology, Software, Writing – original draft, Visualization, Funding acquisition, Supervision, Conceptualization, Project administration. GT: Investigation, Methodology, Software, Writing – original draft, Visualization, Funding acquisition, Supervision, Conceptualization, Project administration. AY: Investigation, Methodology, Software, Writing – original draft, Visualization, Funding acquisition, Supervision, Conceptualization, Project administration.

## Footnotes

^1^We use post-training quantization only for this task because we could not download the training dataset.

^2^Here, the number “5” refers to the number of anchor boxes. The “3” represents the number of classes, which are cars, pedestrians, and two-wheelers. The “4” indicates the attributes of the corresponding anchor box, while the last “1” channel signifies the confidence of prediction for the corresponding anchor box.
